# Studies on genetic diversity, gene flow and landscape genetic in *Avicennia marina:* Spatial PCA, Random Forest, and phylogeography approaches

**DOI:** 10.1186/s12870-023-04475-6

**Published:** 2023-10-03

**Authors:** Laleh Malekmohammadi, Masoud Sheidai, Farrokh Ghahremaninejad, Afshin Danehkar, Fahimeh Koohdar

**Affiliations:** 1 Department of Plant Sciences and Biotechnology, Faculty of Life Sciences and Biotechnology, Shahid Beheshti University, Tehran, Iran; 2 Department of Plant Sciences, Faculty of Biological Sciences, Kharazmi University, Tehran, Iran; 3 Department of Environmental Sciences, Faculty of Natural Resources, University of Tehran, Karaj, Iran

**Keywords:** *Avicennia marina*, Population genetics, Spatial analyses, Migration, Adaptive genetic regions

## Abstract

Mangrove forests grow in coastal areas, lagoons, estuaries, and deltas and form the main vegetation in tidal and saline wetlands. Due to the mankind activities and also changes in climate, these forests face degradations and probably extinction in some areas. *Avicennia marina* is one of the most distributed mangrove species throughout the world. The populations of *A. marina* occur in a limited region in southern parts of Iran. Very few genetic and spatial analyses are available on these plants from our country. Therefore, the present study was planned to provide detailed information on *Avicennia marina* populations with regard to genetic diversity, gene flow versus genetic isolation, effects of spatial variables on connectivity and structuring the genetic content of trees populations and also identifying adaptive genetic regions in respond too spatial variables. We used SCoT molecular markers for genetic analyses and utilized different computational approaches for population genetics and landscapes analyses. The results of present study showed a low to moderate genetic diversity in the studied populations and presence of significant Fst values among them. Genetic fragmentation was also observed within each province studied. A limited gene flow was noticed among neighboring populations within a particular province. One population was almost completely isolated from the gene flow with other populations and had peculiar genetic content.Spatial PCA analysis revealed both significant global and local genetic structuring in the studied populations. Spatial variables like humidity, longitude and altitude were the most important spatial features affecting genetic structure in these populations.

## Introduction

Mangroves are very specialized plants and have adapted to unfavorable environmental conditions, strong tides, high salinity, high temperature, strong winds and anaerobic conditions in the habitat. In such conditions, most of the plant species of dry habitats are not able to adapt. These trees have unique physio-morphological adaptations and tolerance to hyper-saline environments, tidal cycles and soil chemistry [1, 2].

The most-extensive mangrove areas are situated in Asia (42%), Africa (20%), North and Central America (15%), Oceania (12%), followed by South America (11%) [3]. The mangrove species occupying different environmental gradients in a habitat serve collectively for land building, coastal protection, water quality improvement, phytoremediation, carbon sequestration, breeding or nursery ground to various aquatic and terrestrial fauna [4].

Approximately only 6.9% of world’s mangrove areas are protected under the International Union for Conservation of Nature (IUCN) program [5]. These mangrove communities are vulnerable to threats, mainly due to human impact through coastal construction, industrial pollution, littering, loss of water quality, and the development of fisheries. In addition, in some areas, natural disasters, such as earthquakes, tsunamis, coastal erosion, and climate change, threaten mangroves [6–8].

**Fig. 1:**
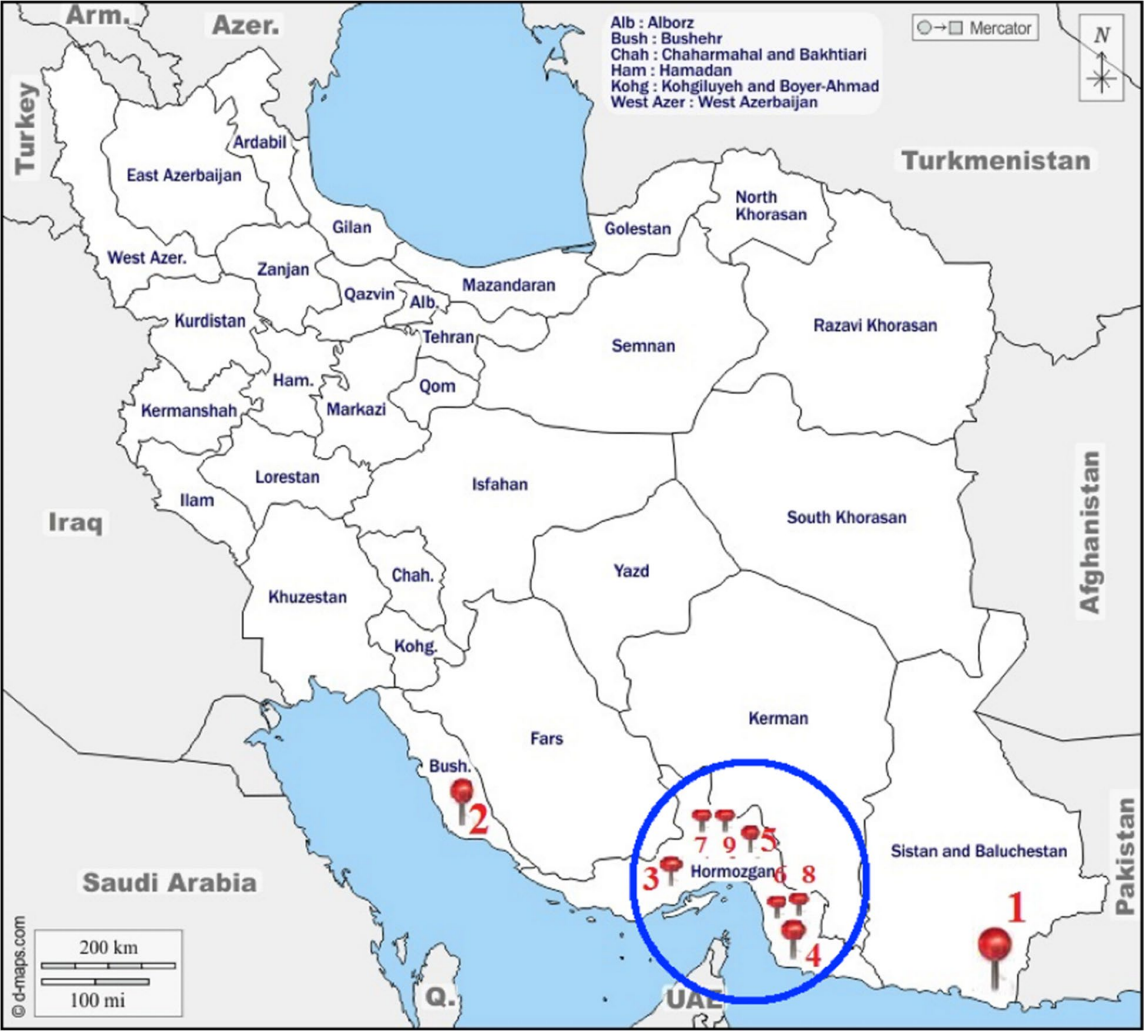
Distribution map of *Avicennia marina* in Iran (Populations 1–9 are according to Table 1). It shows the diverse nature of land cover of the country

*Avicennia marina* (Forsk.) Vierh., is an important mangrove species which can grow and reproduce across a wide range of climatic, saline and tidal conditions. However, its growth is only confined to the southern part of Iran where in comprises a limited number of geographical populations (Fig. 1).

Contemporary mangrove forest residence is based on their genetic connectivity, which in turn is the result of its propagules dispersal. The presence of a suitable environment can improve mangrove subsequent establishment, and persistence. Dispersal of the mangrove seed/propagules may occur in both short and long-distances due to tidal action (hydrochory) and therefore, they may grow either close to the mother tree or in other suitable locations. Therefore, the barriers against the water current or circulation patterns are important for mangrove species’ abundance and distribution [9]. Moreover, the mangrove propagules do not have a dormant stage and their dispersal is also affected by factors such as buoyancy, propagule viability and timely establishment [10]. Therefore, since clonal growth and vegetative dispersal are absent in mangroves, the persistence of the populations depends merely on the formation, release, distribution and establishment of propagules. After establishment, and before reaching sexual maturity, propagules and seedlings are subject to predation, environmental factors affecting early growth and anthropogenic pressure [11].

Population and landscape genetic studies provide data on patterns of gene flow and connectivity among populations and have become an important basis for planning conservation management [12].

Mangrove populations can face environmental changes if they contain an adequate genetic variability. Gene flow and population connectivity may act as a source for providing genetic material for persistence of these populations. With increase in pressures on natural populations due to anthropogenic and climate disturbances, the knowledge of population connectivity becomes vital for identifying vulnerable Mangrove populations and informing management actions at appropriate spatial scales.

The mountainous rims which surround the high interior basins of Iran, and also presence of latitudinal range result in a range of climate change in from arid to sub-tropical across the country. For example, the eastern part of the country is covered by deserts but the northern regions that are covered by the Alborz mountain chain, contains dense forests in the vicinity of the Caspian Sea. The Zagros Mountain is located in the country’s western part and extends from north to south. The Persian Gulf and the Oman Sea coastlines form the country’s southern border.

Population and landscape genetics studies provide important data on genetic structure of species by identifying the gene flow corridor versus the populations with low genetic variability. These data may be used in planning conservation programs for a specific species [13].

These two disciplines combine genetic structure data, landscape ecology, and spatial statistics to investigate the relative influence of landscape and environmental features on gene flow, genetic discontinuities and genetic population structure [14].

Population and landscape genetics studies utilize different molecular markers and computational approaches to produce related data. Molecular markers used are SNPs (Single nucleotide polymorphism), SSRs (Simple sequence repasts), AFLP (Amplified fragments length polymorphism), and SCoT markers (Start codon targeted), etc.

The spatial methods include univariate to multivariate analysis and use multiple regression methods to Bayesian approaches, and data mining analyses [13]. to investigate the presence of spatial autocorrelation, and to identify the effect of continuous variables and gradients on genetic diversity. Similarly, population genetics approaches investigate the genetic diversity, and gene flow, and tries to identify spatial genetic discontinuities. Moreover, phylogeography, and dispersal route analysis, are used to illustrate the pattern of gene dispersal [13].

Identification of potentially adaptive genetic regions or alleles in different local populations is also an important step in these studies [15]. Therefore, due to importance of *Avicennia marina* plants in Iran, the present study was performed based on both population genetic analysis and landscape investigation by spatial principal components (sPCA) analysis, with the following aims: 1- Provide data on genetic structure, and genetic diversity of *Avicennia marina* populations of Iran. 2- Produce data on the genetic discontinuity of these populations versus gene flow. 3- To identify gene flow corridor in southern part of the country and also to illustrate the path of gene migration among different populations. 4—To analyses spatial variables and their effects on spatial genetic structuring of *A. marina* populations, and 5- Identify potentially adaptive genetic regions related to local environmental conditions.

## Material and methods

We used SCoT (Start codon targeted) molecular markers which are suitable molecular marker for both population genetics and landscape genetic investigations [15]. These molecular markers are highly reproducible and occur in different regions of the nuclear genome. Moreover, they show bias toward candidate functional genes, and have been developed based on short conserved sequences of start codons in plant genes, which in turn gives the advantages of no requirement for genomic information [16].

We used different approaches for populations and landscape genetics analyses, due to computation complexity of analytical approaches and a likelihood of difference in some parts of the results obtained.

The analytical methods used in population genetic study ranged from DCA (Dentrented correspondence analysis), DAPC (Discriminant analysis of principal components), RDA (Redundency analysis), and CCA (Canonical correspondence analysis). We used RASP (Reconstruction of ancestral distribution areas), for phylogeography analysis.

Similarly, in landscape genetic studies, we used spatial principal components (sPCA), as it can analyze data in a reduced space and can be used for co-dominant markers as well as presence / absence data as is the case in SCoT molecular markers [17]. This method also carries out Moran and Mantel tests to reveal spatial autocorrelation and test for the occurrence of isolation by distance (IBD).

We also used random forest machine learning approach to identify the importance of spatial and geographical variables on genetic diversity.

## Plant samples studied

In total 119 plants growing in nine geographical populations were studied for both populations genetics as well as landscape genetic studies (Table 1 and Fig. 1).

**Table 1:** Populations and their geographic coordinates

Number of Locality population	Latitude Longitude	Humidity	Rain	Temperature	Number of Samples	
1 Sistan-balochestan	252927	606496	69	124	25.5	15
2 Bushehr	280639	514752	56	215	35	18
3 Hormozgan	265735	555857	60	171	27	19
4 Hormozgan	256577	577857	55	120	25	10
5 Hormozgan	271372	570675	68.5	190	27.5	18
6 Hormozgan	265165	571018	65	150	29	10
7 Hormozgan	267578	557284	52	170	28	9
8 Hormozgan	265165	571018	68	170	28	4
9 Hormozgan	269039	557667	60	115	21	16

*Avicennia marina* leaves were collected from nine populations across mangrove forests in different coastal areas of Iran, including three provinces namely Hormozgan, Bushehr, and Sistan –balochestan, between 2021 and 2022. Populations were sampled based on the accessibility of different habitats and morphological characteristics. According to the special distribution and the nature of the location of mangrove trees in the regions the study was conducted using satellite images and topographical maps, as well as with the help and guidance of local people.

### Molecular studies

#### SCoT assay

The leaf samples collected for genomic DNA extraction were transferred to the laboratory on frozen dry ice and were stored at –80 °C. Cetyltrimethyl-ammonium bromide -activated charcoal protocol (CTAB) was applied to extract the genomic DNA. The extraction was done by activating charcoal for binding of polyphenolics during extraction. DNA isolation was boosted without the interference of impurities [18].

The extracted DNA was examined in terms of quality by running on 0.8% agarose [19]. In this research, four sequences of ScoT primers were used. The polymerase chain reaction (PCR) was set in a final volume of 26 µl, including 14 µl of Master Mix 2x, three microliters of genomic DNA, one microliter of each primer with a concentration of ten picomoles per microliter, and eight microliters of sterile distilled water.

Four primers (SCOT1, SCOT2, SCOT5, and SCOT9) were selected based on Collard and Mackill [20]. The primer sequences are: SCoT1: CAACAATGGCTACCACCA, SCoT2: CAACAATGGCTACCACCC, SCoT 5: CAACAATGGCTACCACGA, SCoT9: CAACAATGGCTACCAGCA.

The amplification reactions were performed in Techne thermocycler (Germany) with the following program: 4 min at 94 ºC, 35 cycles of 1 min at 94 ºC, 1 min at 50–60 ºC and 2 min at 72 ºC and a final cycle of 5min at 72 ºC. The amplification products were visualized by running on 2% agarose gel, stained with cyber green (Powerload, Kosar Co. Iran). The fragment size was estimated by using a 100 bp molecular size ladder (Fermentas, Germany).

### Data analysis

#### Genetic diversity analyses

The SCoT bands obtained were treated as binary characters and coded accordingly (presence = 1, absence = 0). For genetic diversity analyses,we estimated population genetic diversity parameters as performed in GenAlex ver.6 [21]. Similarly, the genetic differentiation of the populations was studied by calculating Fsts from AMOVA (Analysis of molecular variance), as implemented in Gen Alex software [21].

Grouping of the populations based on genetic data was performed by both clustering and ordination methods. We used both Jaccard and Dice genetic similarity index for these analyses, as they are appropriate for binary data as in SCoT markers. We used PAST program ver.4 for these analyses [22].

DAPC method was used to show genetic admixture and assignment of individuals to different genetic groups. This was done in adegenet package of R. 4.1 [17].

Association between genetic data and geographical variables was determined by RDA and CCA methods by 999 permutations as performed in PAST program ver.4. [22]. Similarly, LFMM (Latent factor mixed model) was used to identify the SCoT loci with significant association with geographical variables. This was done after utilizing FDR (False determined ration), as performed in LFMM package in R. ver.4.1 [23].

CCA (Canonical correspondence analysis), is based on the regression of the SNPs and ecological features. It uses an approach similar to principal components analysis (PCA). However, in PCA, we have a maximized variance of data, while CCA tries to maximize the association of data (SNPs), to geographical variables [24].

RDA is a form of constrained ordination that suits for genomic data sets, where we are interested in understanding how the multivariate environmental factors shape the patterns of genomic composition across geographical areas. RDA is a direct gradient analysis technique, which summarizes linear relationships between components of response variables that are "redundant" with (i.e. "explained" by) a set of explanatory variables. It is based on multivariate regression [25].

LFMM is a Bayesian method for testing associations between loci and geographical gradients using latent factor mixed models. It performs a regression analysis in which the confounding variables are modeled with unobserved (latent) factors. The program estimates correlations between geographical and ecological variables and allelic frequencies, and simultaneously infers the background levels of population structure [23].

### Spatial analyses

#### sPCA

We used spatial PCA method (sPCA) to investigate the spatial pattern of genetic variability based on allelic frequency data of individuals or populations [17]. This approach is independent of presumed Hardy–Weinberg expectations or linkage equilibrium among loci and uses statistical (Monte Carlo) tests to partition the spatial structure into random, local, and global variance patterns, where local patterns are taken to relate to highly negative spatial autocorrelation and global patterns are taken to relate to highly positive spatial autocorrelation.

The Spatial autocorrelation is measured using Moran’s I [26], that is incorporated within the sPCA algorithm. It differentiates between global structures (patches, clines and intermediates) and the local ones (which represents strong genetic differences between neighbors) and from random noise [17]. These analyses were performed in Adegenet package in R 4.1 [27].

### Random forest (RF) analysis

RF (Random Forest) is a machine learning method which is used in spatial analysis with the aim to illustrate the importance of spatial factors affecting genetic differentiation (Fst value) in the studied populations. This is a supervised machine learning algorithm that is used in classification and Regression problems. It builds decision trees on different samples and takes their majority vote for classification and average in case of regression [28]. This approach is consists of many decision trees and uses bagging and feature randomness when building each individual tree to try to create an uncorrelated forest of trees whose prediction by committee is more accurate than that of any individual tree.

Random Forests illustrates the feature importance, based on calculated Gini Index. A lower value of this index shows the importance of that variable and reveal association between genetic data and landscape features [29]. RF analysis was performed in random Forest package of R. 4.1, with 500 bootstraps [27].

### Phylogeography

In phylogenetic approach we used a maximum likelihood phylogenetic tree obtained from SCoT molecular markers as the base tree which was later on used for reconstruction of ancestral area analysis and connectivity as performed in RASP ver. 4 [30]. We used both MCMC binary as well as S-DIVA methods for construction of the phytogeography tree.

## Results

**Fig. 2:**
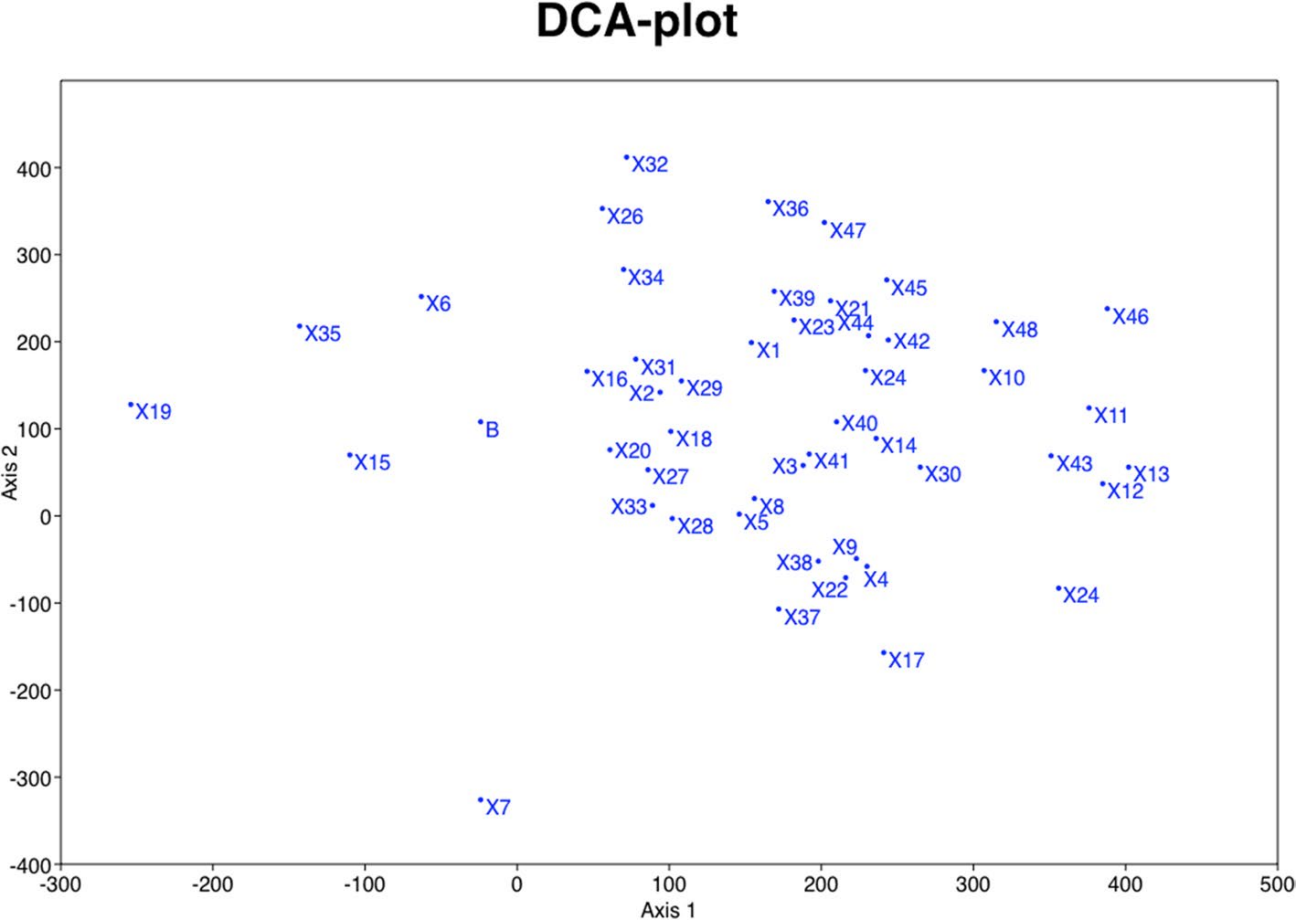
DCA plot showing a scattered pattern of distribution for the SCoT markers in different parts of the genome

We obtained 48 distinct and stable SCoT loci for population genetic analyses, which were used in sPCA analysis. DCA plot (Fig. 2), showed that SCoT loci are distributed in different parts of the genome and are not linked, therefore are suitable molecular markers for population genetic studies.

Details of SCoT bands obtained in 119 samples of nine *Avicennia marina* geographical populations are provided in (Table 2).

**Table 2:** SCoT bands’ distribution among geographical populations of *Avicennia marina* (The names of populations are provided in Table 1)

Number of Population	1	2	3	4	5	6	7	8	9
Number of Bands	39	29	24	20	28	27	24	21	17
Number of Bands Bands Freq. > = 5%	39	29	24	20	28	27	24	21	17
Number of Bands Private Bands	5	1	0	0	0	1	0	0	0
Number of Bands LComm Bands (< = 25%)	3	2	0	0	1	0	1	1	0
Number of Bands LComm Bands (< = 50%)	13	7	5	4	9	8	5	4	4

No. Bands = No. of Different Bands

No. Bands Freq. >  = 5% = No. of Different Bands with a Frequency >  = 5%

No. Private Bands = No. of Bands Unique to a Single Population

No. L Comm Bands (< = 25%) = No. of Locally Common Bands (Freq. >  = 5%) Found in 25% or Fewer Populations

No. LComm Bands (< = 50%) = No. of Locally Common Bands (Freq. >  = 5%) Found in 50% or Fewer Populations

A few private bands were obtained in populations 1, 2, and 6. These private bands (genetic regions) may help the local adaptation of these plants

### Genetic diversity parameters

Genetic diversity parameters in the studied populations are provided in Table 3. In general, a low to moderate degree of genetic variability ranging from 25.00% in pop 8 to 66.67% in pop 1, is present in *A. marina* populations studied. Among the studied populations, pop 1 has a higher magnitude of gene diversity (He = 0.20), and percentage of genetic polymorphism (%P = 66.67%) compared to the other studied populations.

**Table 3:** Genetic diversity parameters determined in *A. marina* populations (The names of populations are provided in Table 1)

Number of Population	N	Ne	I	He	uHe	%P
1	15.000	1.346	0.320	0.209	0.217	66.67%
2	18.000	1.210	0.195	0.127	0.130	43.75%
3	19.000	1.200	0.192	0.124	0.127	43.75%
4	10.000	1.224	0.182	0.123	0.130	35.42%
5	18.000	1.319	0.280	0.187	0.192	56.25%
6	10.000	1.429	0.334	0.232	0.245	54.17%
7	9.000	1.292	0.253	0.170	0.180	47.92%
8	4.000	1.118	0.120	0.077	0.088	25.00%
9	16.000	1.157	0.133	0.089	0.092	27.08%

*N* Number of plants studied, *Ne* Number of effective alleles, *I* Shannon information index, *He* Gene diversity, *uHe* Unbiassed gene diversity, *%P* Percentage of genetic polymorphism

The Nie’s Gst analysis of SCoT loci with regard to gene flow (Nm) and genetic differentiation (Gst) is provided in (Table 4) (Only important loci tree provided).

**Table 4:** Nm (Gene flow), versus genetic differentiation (Gst) of the studied SCoT loci in *Avicennia marina* populations studied

Locus	Sample Size	Ht	Hs	Gst	Nm
Locus7	119	0.1071	0.0983	0.0823	5.5737
Locus10	119	0.2417	0.0508	0.7900	0.1329
Locus 13	119	0.0314	0.0273	0.1298	3.3532
Locus14	119	0.1695	0.1377	0.1878	2.1624
Locus 17	119	0.1063	0.0927	0.1280	3.4071
Locus 19	119	0.2864	0.0779	0.7280	0.1869
Locus 21	119	0.0542	0.0495	0.0874	5.2197
Locus23	119	0.0936	0.0794	0.1516	2.7974
Locus24	119	0.0399	0.0333	0.1665	2.5028
Locus25	119	0.0075	0.0073	0.0303	16.0264
Locus26	119	0.0593	0.0542	0.0863	5.2957
Locus27	119	0.2765	0.2120	0.2332	1.6439
Locus30	119	0.0200	0.0193	0.0388	12.3881
Locus42	119	0.4346	0.1067	0.7544	0.1628
Locus48	119	0.0484	0.0442	0.0852	5.3717
Mean	119	0.2569	0.1487	0.4211	0.6874

*Nm* estimate of gene flow from Gst or Gcs, *Hs* Sub Populations Heterozygosity, *Ht* Total Heterozygosity

Some of the SCoT loci had a good magnitude of Nm and are shared common alleles among *Avicennia marina* populations. These loci have Nm value > 2 (indicated in Table 4). Similarly, a few loci have high Gst value (> 0.7), which indicates they are private bands (discriminating loci), in one or a few populations. In general, the mean Nm value = 0.6 indicates a moderate gene flow among the studied populations.

The results show that there is a genetic diversity moderate(25–66 percent of polymorphism)in the studied populations. AMOVA revealed a significant difference among the studied populations. About 48% of total genetic difference was among populations, while about 52% was within population genetic variability. These results indicate that *Avicennia* populations not only differ genetically from each other, but each local populations contains a good level of genetic variation. AMOVA-paired revealed significant genetic differences (*P* < 0.01), among the studied populations, which shows in spite of potential across populations gene flow, they still have a mean genetic distinctness that differentiate them from each other.

### Grouping of the studied populations based on genetic data

The Nei’s genetic distance of the studied populations is provided in (Table 5).

**Table 5:** Nei genetic distance (bellow diagonal) versus genetic similarity (above diagonal) determined among *A. marina* populations (The names of populations are provided in Table 1)

Number of population	1	2	3	4	5	6	7	8	9
1		0.8395	0.8237	0.8185	0.8468	0.8183	0.7540	0.7068	0.7565
2	0.1750		0.8470	0.8164	0.8440	0.8363	0.8164	0.7626	0.8204
3	0.1939	0.1660		0.9740	0.9479	0.8749	0.9009	0.8329	0.9072
4	0.2003	0.2028	0.0263		0.9494	0.8852	0.9092	0.8367	0.8885
5	0.1663	0.1696	0.0535	0.0520		0.9038	0.8942	0.8024	0.8739
6	0.2005	0.1788	0.1336	0.1220	0.1011		0.9352	0.8575	0.8789
7	0.2824	0.2029	0.1044	0.0952	0.1118	0.0670		0.8664	0.8930
8	0.3470	0.2710	0.1829	0.1782	0.2201	0.1537	0.1435		0.9607
9	0.2791	0.1979	0.0974	0.1183	0.1348	0.1291	0.1132	0.0401	

In general, a high degree of genetic similarity was observed among the studied populations, which ranged from 0.70 to 0.96.

**Fig. 3:**
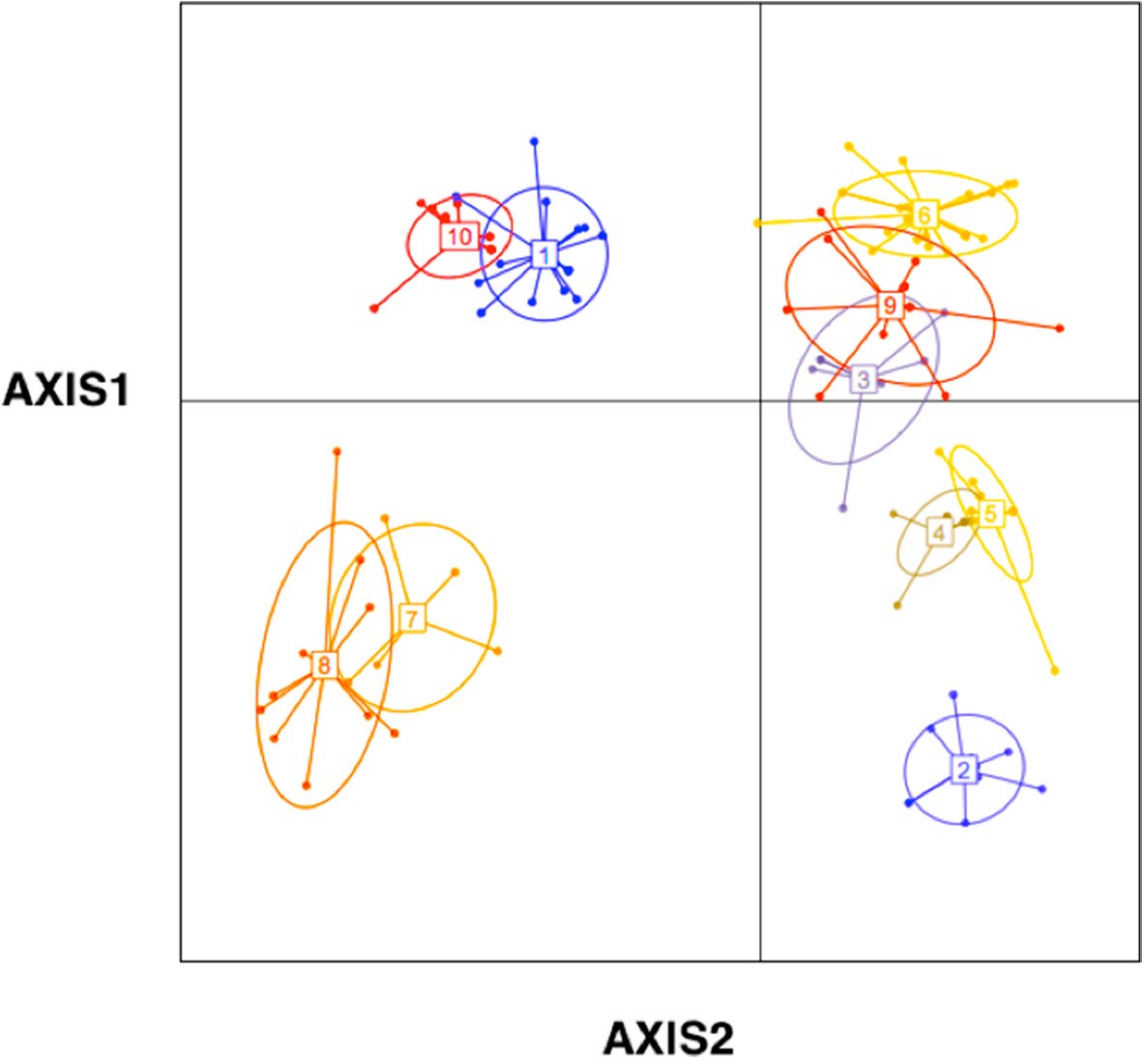
Genetic grouping of *A. marina* populations by LDA plot shows four major genetic groups

**Fig. 4:**
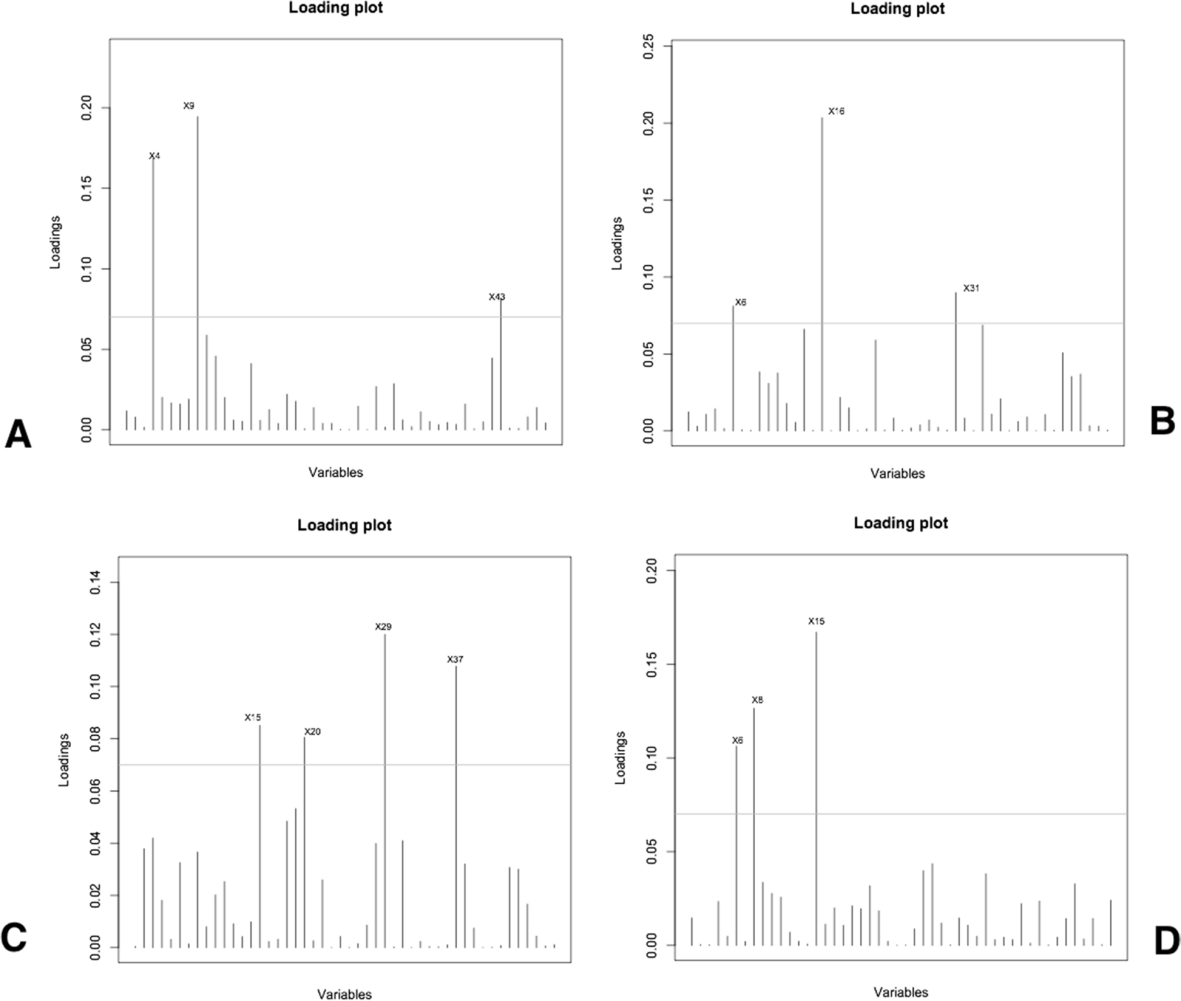
Differentiating SCoT loci identified by LDA loadings

The highest degree of genetic distance (0.34), occurred between populations 1 and 8, followed by populations 1 and 9 (0.279). Grouping of these populations based on genetic data by LDA method is provided in (Fig. 3). The primitive analysis showed that the first three LDA axes comprise almost 90% of total discriminating values (Fst values) among the studied populations. Grouping of the populations based on the first two LDA axes, produced four major genetic group (Fig. 4).

Populations 1 and 2 are placed far from the other studied populations due to genetic difference. A close genetic affinity is observed among populations 3, 6, 9, and 4, due to presence of common alleles. Similarly, populations 7 and 8 show genetic affinity and are placed together.

It is interesting to see that, another extra genetic group (labeled 10, in Fig. 3), has been recognized by LDA plot. This shows that a genetic fragmentation has occurred within population 1 (Sistan-o-Balouchistan province).

LDA identified some of SCoT loci, which differentiate the studied populations (Fig. 4). These are SCoT loci 4, 9, 43, 6, 8, 15, 16, 31. 15, 20, 29, and 37.

### Potentially adaptive genetic regions

**Fig. 5:**
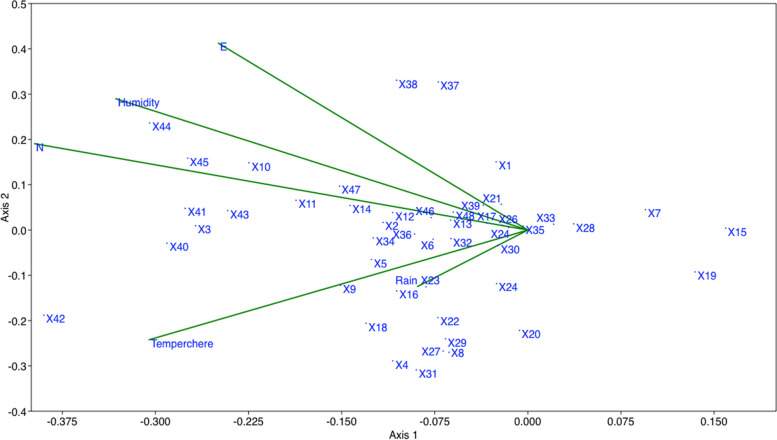
RDA plot of SCoT loci showing association of several loci with environmental variables

Both RDA and CCA plots after 999 permutations produced a similar and significant association (*p* < 0.01) between SCoT loci obtained and geographical and environmental variables studied. Therefore, only RDA plot is presented in Fig. 5. SCoT loci 3, 16, 23, 40–45 are among potentially adaptive loci identified by RDA.

**Fig. 6:**
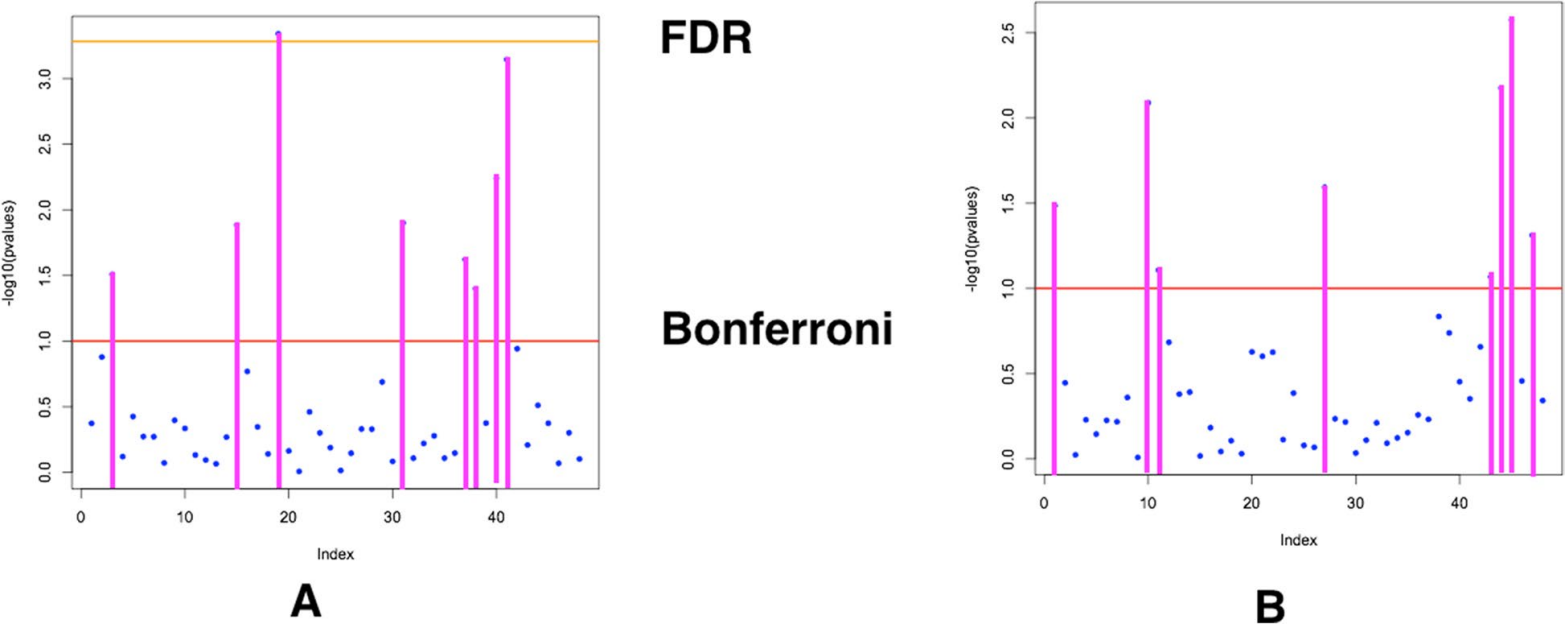
Manhattan plots of LFMM analysis, show SCoT loci associated with longitude and latitude (**A**), and humidity and temperature (**B**)

Manhattan plots of LFMM analysis revealed that SCoT loci 3, 15, 19, 31, 37, 38, 40, and 41, are significantly associated with longitude and latitude (Fig. 6A). Similarly, a significant association was obtained for SCoT loci 1, 10, 11, 27, 43–45, with humidity and temperature (Fig. 6B).

**Fig. 7:**
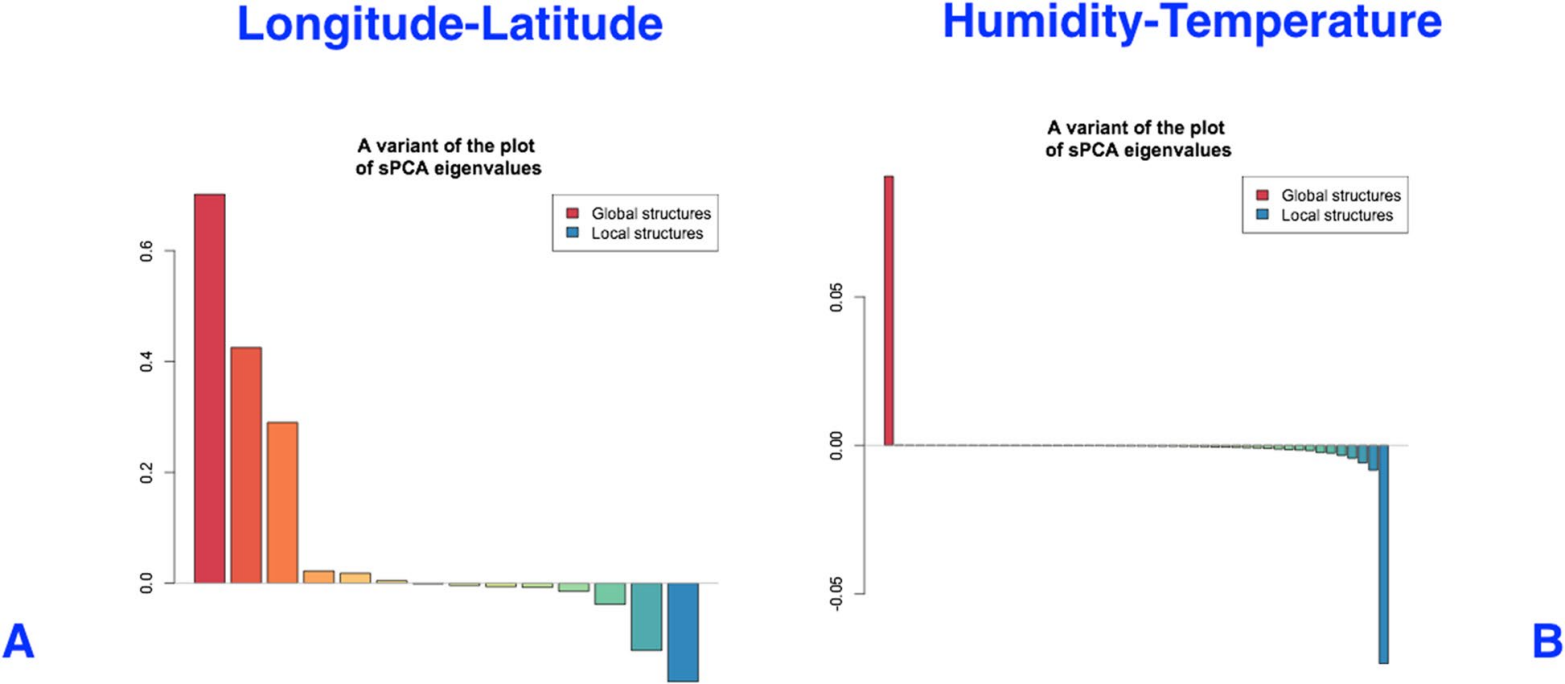
sPCA plots showing eigen values and connectivity of plants based on geographical variables of longitude and Latitude (**A**) and humidity and temperature (**B**)

The results of sPCA analysis for both pairs of longitude and latitude, as well as humidity and temperature show significant effects of these spatial variables on the genotypes studied. We obtained both significant positive and negative Eigen values for all these variables (Fig. 7), showing that *A. marina* populations are genetically structured by both large scale and local spatial features.

**Fig. 8:**
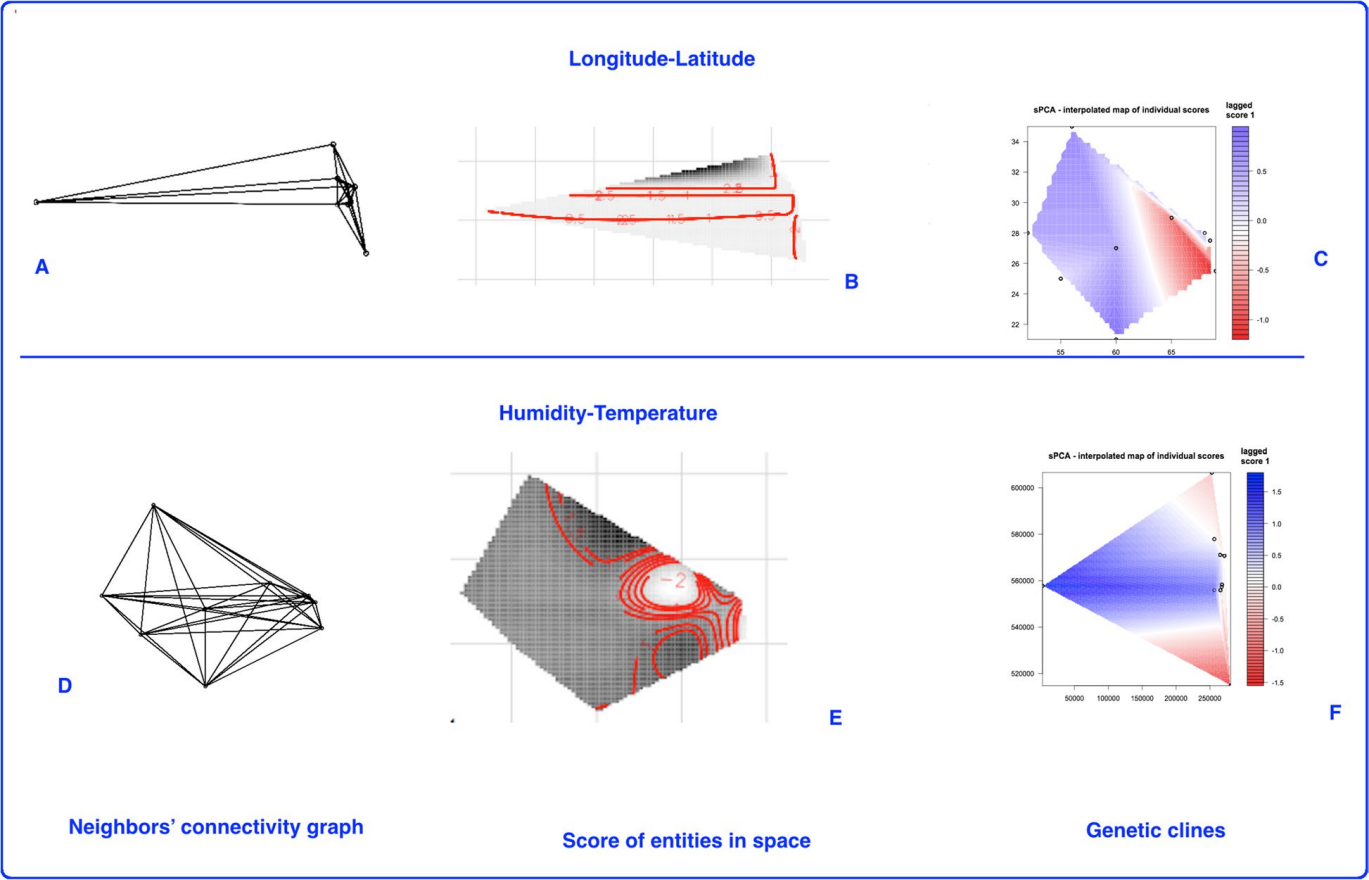
Plots of sPCA showing neighbors’ connectivity, score of entities in space and genetic clines in respond to longitude and latitude (**A**-**C**), and humidity and temperature (**D**-**F**). These figures reveal genetic isolation along with spatial variables studied

IBD analysis and Morans’ I index after 999 times permutations produced significant results for these variables (*p* < 0.01). Significant Mantel test and IBD indicate that with increase in geographical distance of *A. marina* populations, the genetic difference increases between them. Similarly, as presented in (Fig. 4), Morans’ I index was significant for both global and local spatial features studied. Moreover, the global and local tests (m-tests in sPCA package), produced significant *p* < 0.01, after 999 permutations. Therefore, both global and local structures affect the genetic distribution of *A. marina* populations. The neighbors’ connectivity graph and the scores of entities in space, as well as genetic clines of the populations are provided in (Fig. 8). These figures also support populations’ genetic isolation in space.

### Random forest results

We used Fst values of the studied populations and categorized them in distinct levels from A-F classes, depending on degree of differentiation, i.e. higher value of Fst was F, and decreasing down-wards to A class with the lowest Fst value (Table 6).

**Table 6:** Fst values of the studied populations

Stat	Value	P(rand > = data)
PhiPT	0.449	0.001
PhiPT max	0.831	-
Phi'PT	0.540	-

These Fst values were studied with respect to spatial features to identify their importance. RF analysis with 500 bootstraps revealed 100 percent accuracy for train data, and 80% for predictive data, which shows a good fit of results to the model provided. Based on the Ginni, the importance of the studied feature starts with latitude (Ginni value = 8.02), followed by humidity (Ginni value = 8.12). The lowest importance value was obtained for temperature (Ginni value = 20.00), followed by rain (Ginni value = 15.30).

**Fig. 9:**
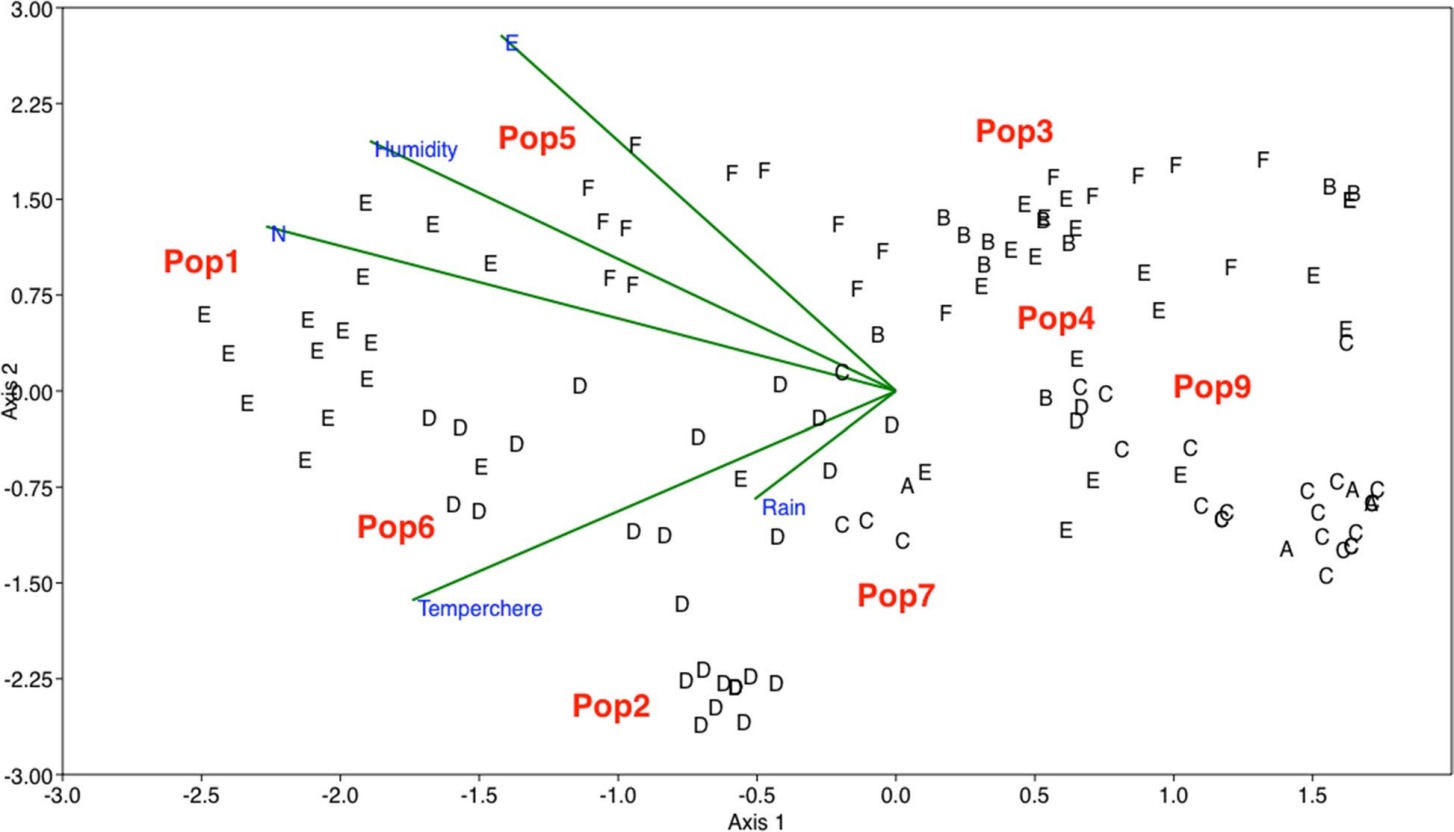
RDA plot of spatial features and Fst values in *A. marina* populations, showing that the higher Fst values are associated with latitude, longitude and humidity. (Populations 1–9 are as in Table 1)

RDA plot constructed based on both Fst values and spatial variables (Fig. 9), supports the variables identified important by the RF method, and shows distribution of geographical populations along with these spatial variables.

RDA analysis produced significant results (*p* < 0.01), with two components. This indicates a strong correlation between spatial features studied and Fst values. It also shows that the higher value of Fst classes (F and E classes), are structured by longitude, altitude and humidity.In contrast, temperature and rainfall mainly structure the populations with a lower Fst class (D classes). These results indicate the complex effects of spatial features on structuring genetic content of *Avicennia marina* populations.

**Fig. 10:**
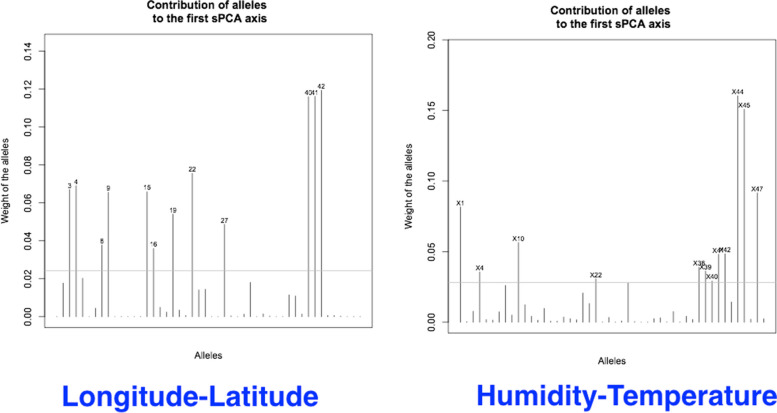
Contributing SCoT loci to spatial distribution and structuring of *A. marina* plants

The contribution of SCoT loci to the spatial distribution of plants studied is printed in (Fig. 10). These results are very similar to the RDA and CCA analyses performed to identify potentially adaptive SCoT loci presented before. Therefore, these genetic regions are also identified as most contributing genetic regions to the spatial distribution of *A. marina* plants.

### Gene flow corridor and gene migration route

**Fig. 11:**
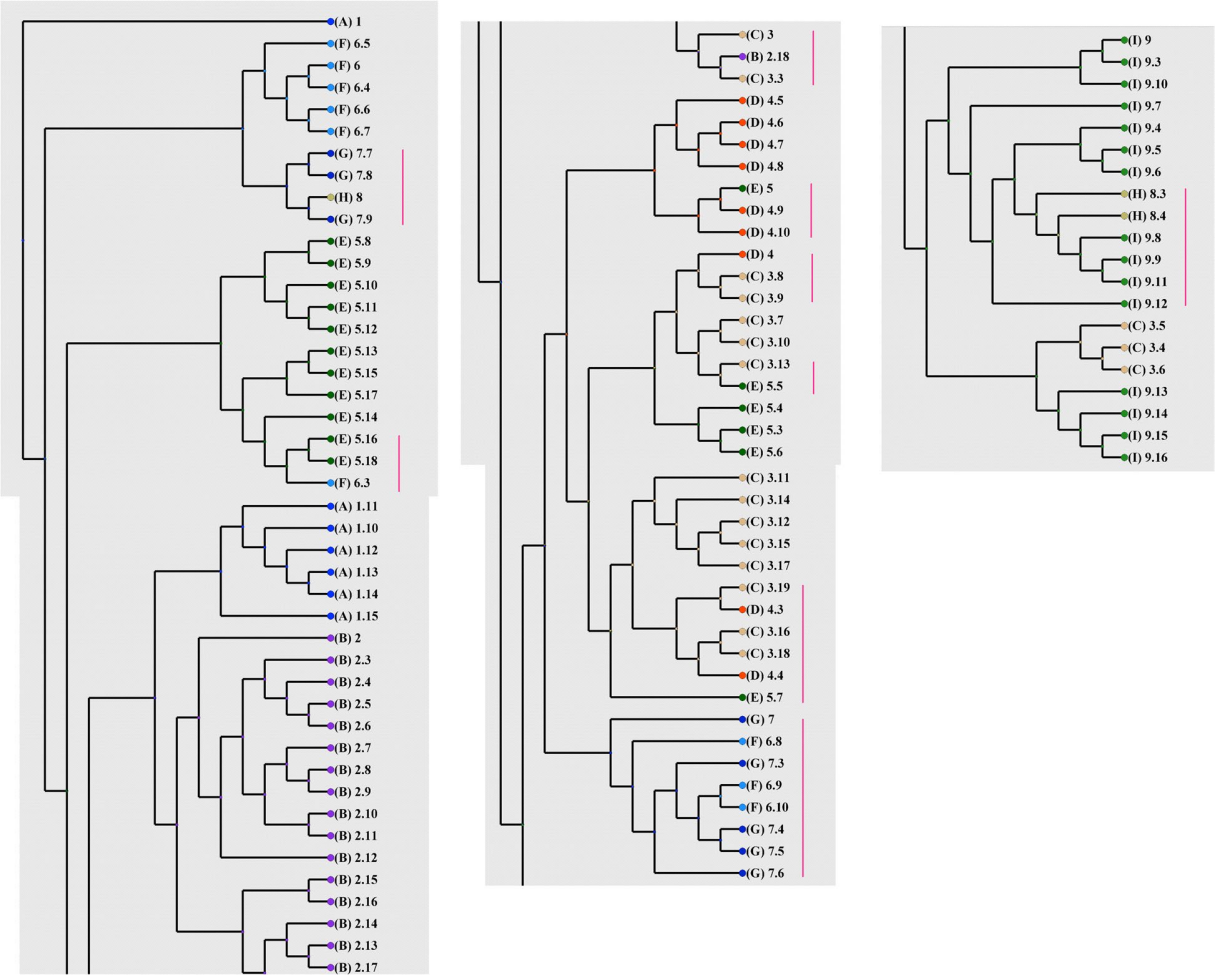
RASP phylogeography tree showing gene flow and migration mainly among populations of Hormozgan province (pops 3–8). (Pops 1–9 are according to Table 1)

**Fig.12:**
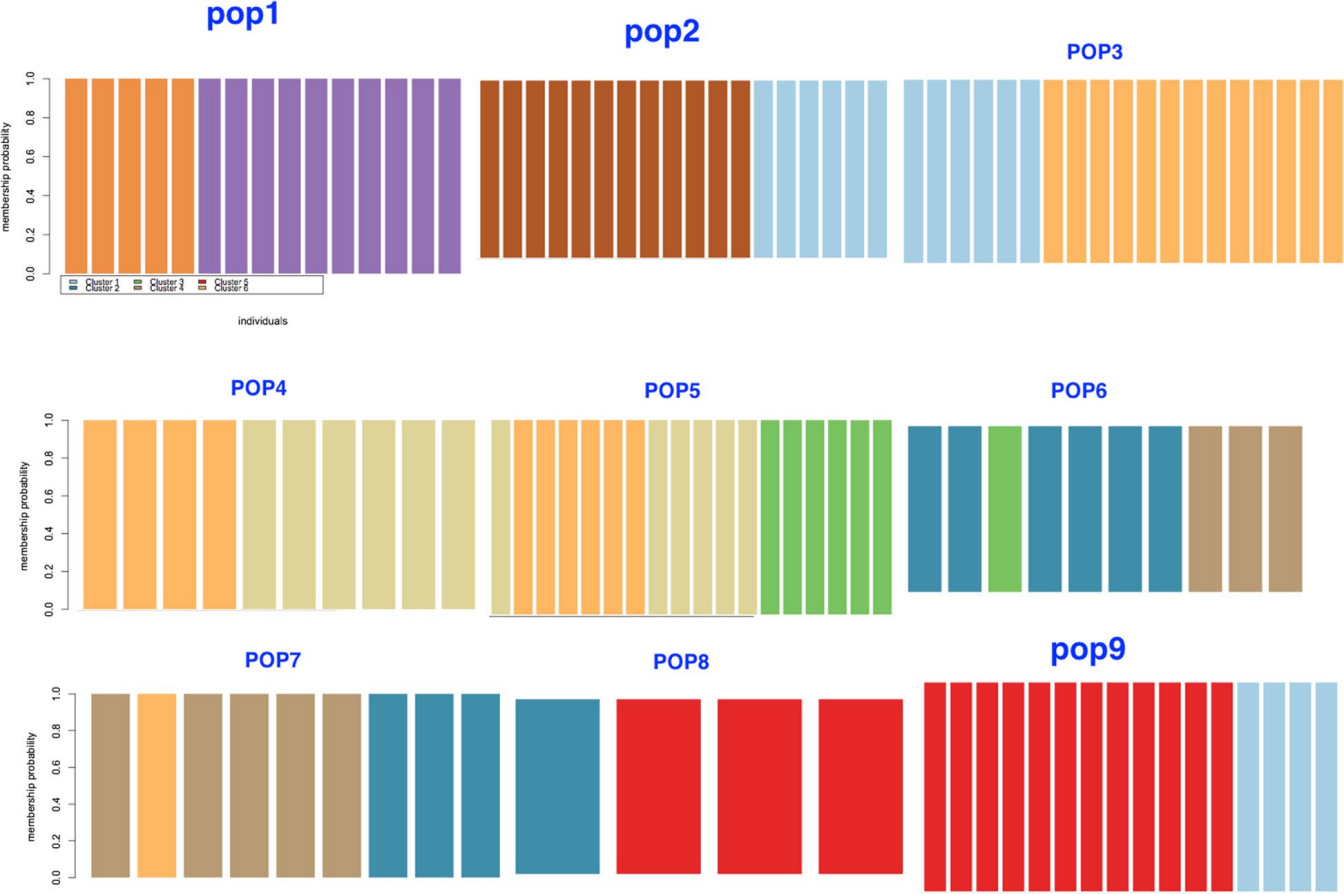
Assignment test of DAPC showing genetic admixture among *A. marina* populations. (populations 1–9 are as in Table 1)

Phylogeography (Fig. 11) and assignment test (Fig. 12), were performed to study gene flow and genetic admixture between the studied populations and also to identify the gene flow corridor as well as migration route. The results of both analyses reveals that gene flow and admixture occur mainly within populations of Hormozgan province i.e. populations 3–9.

It is also important to mention that these populations differ significantly in their Fst and in spite of gene flow they contained specific genetic content which makes them genetically different.This may be due to spatial local structuring of these populations.

**Fig. 13:**
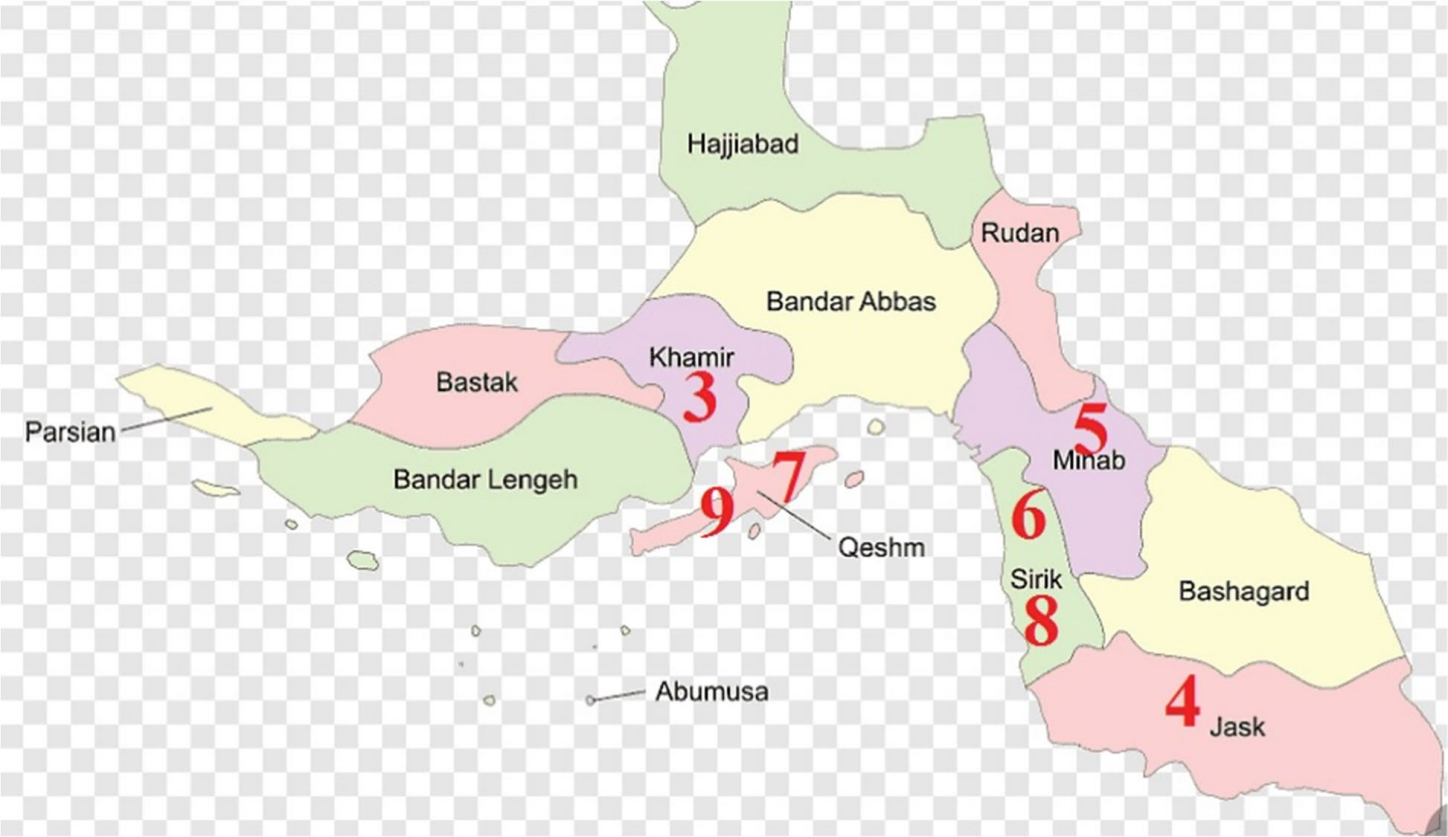
Hormozgan populations show a higher magnitude of gene flow and may be considered as gene flow corridor for *A. marina*

The gene flow corridor has been marked and shown in (Fig. 1) (Circled area), and detail in (Fig. 13). The population 1 is genetically differentiated from the rest of studied populations, but population 2, shows some degree of genetic admixture with population 3. We may therefore consider the Hormozgan province as the corridor of gene flow in *A. marina* populations studied. This area is the central region of the plants geographical distribution, and therefore, it may be concluded that from this central region, gene migration has occurred towards marginal populations (population 2). However, population 1 is genetically differentiated from the other studied populations and shows no genetic admixture with them. Therefore, we may suggest a loss of common shared ancestral alleles in this particular population due to local adaptation/ or selection.we should consider the other areas which are not studied by us as the source of parental alleles for this locality.

## Discussion

### Genetic diversity and connectivity

We obtained almost a low genetic polymorphism in *A. marina* populations ranging from 0.25 to 66%. A similar study performed on genetic diversity of *A. marina* by SSR markers in three populations of Bushehr province of Iran by Valipour Kahrood et al. [31], also reported the level of heterozygosity, overall loci, ranged from 0.451 to 0.667 with and a high inbreeding coefficient.

The reports on genetic diversity of *A. marina* from other parts of the world are also in agreement with results of present study. For example, Maguire et al. [32] studied the level of genetic variation throughout the entire worldwide range of *Avicennia marina* by using micro-satellite markers. They reported that the levels of heterozygosity detected for each population, overall loci, ranged from 0.0 to 0.8, with an average of 0.407, indicating that some populations had little or no genetic variation, whereas others had a large amount. They noticed that populations at the extremes of the distribution range showed reduced levels of heterozygosity, and significant levels of inbreeding, which is in agreement with our results. We observed a unique genetic structure in population 1 (Sistan -balochestan), much different from the other studied populations. This locality is placed far from the other studied localities and showed no signs of genetic admixture. Local genetic structuring by spatial variables was also significant in this region. Therefore, plants in this population may have faced inbreeding to lack of connectivity with the other populations.

In a similar study, Lu et al. [33], investigated the genetic diversity, spatial genetic structure, and mating system of two mangrove species, *Rhizophora apiculata* and *Avicennia marina*, in a heavily disturbed area in Tielu Harbor, Sanya City, Hainan Island, China, by using SSR markers and reported a significant positive Fst values and high levels of inbreeding *A. marina* populations. The reason for the high level of inbreeding in *A. marina* thought to be the habitat degradation and fragmentation, which was also reported for *Avicennia germinans* L. [34].

We considered Hormozgan province with its populations as the corridor of gene flow among the studied populations. We noticed migration and gene flow from this central region to the marginal population of Bushehr (population 2).

In a similar study Rolland et al. [35] used niche modeling and landscape genetics to study local adaptation in alpine plants. They used AFLP (Amplified length polymorphic data) as the molecular marker and showed that some of AFLP loci are adaptive and that some level of shared loci were obtained in central and marginal populations. They also concluded that gene flow occurred from central to the marginal areas and suggest the results are in accord to the models of species range evolution, in which the center of the niche contributes to the emergence of novel adaptive alleles, which diffuse towards niche margins and facilitate niche and range expansion through subsequent local adaptation [35].

The present study showed significant paired Fst and populations’ genetic differentiation. Moreover, a significant global and local spatial structuring prevails on genetic structure of *A. marina* populations studied. This species is the most widely distributed mangrove species worldwide, which is considered due to long distance dispersal (LDD) of its buoyant propagules, coupled with tolerance to a wide range of environmental conditions [12]. However, several studies have reported that LDD in *A. marina* is rare and the majority of propagules disperse less than 1 km from their release point and rarely over 10 km [36].

These results are in accord to the present study findings. Different studies have reported both long-range connection of mangrove trees in relation to ocean currents and direction, especially along same coastlines, and also the occurrence of a stepping-stone model of migration between estuaries [36, 37]. The present study aligns with several studies throughout the world, which shows that. *A. marina* populations are genetically distinct and exhibit a new connectivity level.

Binks et al. [12], suggest that for the management of *A. marina*, these genetically distinct populations and subpopulations should be treated as separate management units. Similarly, it was suggested [33], that for mangrove restoration, it is better to use the propagules produced by local adult trees and also the connectivity among individuals between newly restored mangroves with existing mangroves should be maintained, to minimize the effects of inbreeding on future generations. Furthermore, Salas-Leiva et al. [34], suggest that reforestation using propagules from different populations would improve the maintenance of genetic diversity and the viability of the reforested population in the short and medium term. Such populations are resilient enough to persist and tolerant against the anthropogenically driven habitat degradation and climate change [38, 39].

### Association studies

The present study identified genetic regions by SCoT loci, which are significantly correlated with geographical and landscape variables. These loci were identified by different analytical approaches employed. It seems therefore, using different approaches may improve understanding of associated SNPs or genetic regions with geographical and ecological variables and such a combined data evaluation, give insights into contemporary evolutionary processes, and may explain how environmental factors influence selective and neutral genomic diversity within and among related species or different geographical populations within a single species [40].

Presence of heterogenous environmental conditions bring about changes in the genetic diversity of plant species, which in turn results in local adaptations [15, 40, 41]. Therefore, the studies concerned with the genetic basis of local adaptation and identifying adaptive genetic loci or SNPs can improve the knowledge of the genetic mechanism of local adaptation and probably species diversification within a genus [15].

Identification of important genes with specific functions is a critical task in genetic studies of *A. marina*. In conclusion, we provided novel findings on genetic structure, gene flow and spatial structuring of genetic content in *A. marina* populations. We also identified the genetic regions associated to the geographical, and land scape variables in *A. marina*.

## Data Availability

The datasets analyzed during the current study are available from the corresponding author on reasonable request.
